# Prenatal Care

**Published:** 1919-01

**Authors:** 


Armenian Relief Campaign, February 3-10.

### Prenatal Care.

No greater work could be undertaken by the women's clubs of the country than to secure the services of physicians or ject of prenatal care. There is woeful ignorance upon the subject of childbirth and especially regarding the care that prospective mothers should take of themselves.

Of the 100,000 babies who die every year in this country before they are a year old, nearly half die during their first month of life, Mrs. Max West writes for the press service of the Children's Bureau, United States Department of Labor. In many tiny bodies the flame of life burns so feebly at birth that it is soon snuffed out, because mothers were ill or overworked or underfed during the momentous months before their babies were born. That is why the Children's Bureau of the United States Department of Labor and the Woman's Committee of the Council of National Defense urge that as part of the Children's Year campaign to save 100,000 babies proper instruction and care be made available for all mothers during the critical months before their babies come.

That mothers themselves realize that the care of the baby must start before the baby is born is shown by their eagerness to avail themselves of information concerning the hygiene of maternity. The demand for the pamphlet on Prenatal Care, published by the Children's Bureau, is steadily increasing. In California excerpts from that pamphlet, as well as from the Children's Bureau pamphlet on Infant Care, have been translated into Japanese for the benefit of the Japanese mothers of the Pacific Coast. The prenatal centers that have been established during Children's Year in many towns throughout the country are eagerly sought by mothers who desire advice on how to make child-bearing safe for themselves and for their children.

At least 15,000 mothers are lost to the United States each year from causes—most of them preventable—that are connected with childbearing. They die because they have not known how to protect themselves during pregnancy or because the surgeon is not within reach at the time of confinement or because the doctor or midwife who attends them is careless or ignorant. Many other mothers who survive in spite of improper care are left in a weakened condition, incapable of properly looking after the needs of their families.

This loss, coupled with the loss of thousands of babies, is one that no country can afford to ignore. In England, after four years of war, prenatal care for mothers has greatly increased. Only recently the King has put his signature to a bill that provides Government aid for medical and nursing care for mothers and babies. A similar bill, destined to help mothers of the United States—especially those in rural districts—has been presented in our own Congress.

What may be accomplished by making available for mothers advice and proper care is shown by the record of one prenatal center in the city of Phildelphia. Not one death in childbirth has occurred in three years among the mothers attending the center, and not a case of eclampsia (convulsions of pregnacy). Out of ninety-nine full-time births, ninety-four were living at the end of the first month. All but two were breast-fed.

In New York the Milk Committee has been in touch with more than 3,000 mothers. Only five of these mothers lost their lives from causes connected with childbirth and only eighty-six babies died in the first month of life. These figures show a reduction of 69 per cent in the death rate for mothers and of 28 per cent in the death rate for babies among the supervised cases as compared with the figures for New York in general. One of the largest insurance companies of the country has in the last few years been trying the experiment of sending a nurse to expectant mothers among its policy-holders. In 1916 these nurses visited more than 7,000 cases and the deaths in childbirth among the company's policy-holders decreased from 70.1 per thousand in 1911 to 62.5 in 1916—a decrease of 10.7 per cent.

The provision of such care for all mothers would prevent a great waste to our country—a waste that makes itself felt, not only in mothers and babies that actully die, but in invalid women and puny children destined for lifetime ill-health and inefficiency.

---

"They shall not perish." Give to the Armenian Relief Campaign.

